# *ZmGLP1*, a Germin-like Protein from Maize, Plays an Important Role in the Regulation of Pathogen Resistance

**DOI:** 10.3390/ijms232214316

**Published:** 2022-11-18

**Authors:** Lixue Mao, Lijie Ge, Xinchun Ye, Li Xu, Weina Si, Ting Ding

**Affiliations:** 1 Key Laboratory of Biology and Sustainable Management of Plant Diseases and Pests of Anhui Higher Education Institutes, School of Plant Protection, Anhui Agricultural University, Hefei 230036, China; 2 National Engineering Laboratory of Crop Stress Resistance Breeding, School of Life Sciences, Anhui Agricultural University, Hefei 230036, China

**Keywords:** maize, germin-like protein *ZmGLP1*, Jasmonic acid, hydrogen peroxide, disease resistance

## Abstract

A gene encoding a protein similar to germin-like proteins (GLPs) was obtained from maize (*Zea mays*) and designated as *ZmGLP1*. Based on the *ZmGLP1* conserved domain and phylogenetic status, *ZmGLP1* was grouped into GLP subfamily b and has high similarity to *OsGLP8-14* from *Oryza sativa*. *ZmGLP1* is expressed in different maize tissues during different growth stages and is mainly expressed in the stems and leaves. The induced expression patterns confirmed that *ZmGLP1* is differentially expressed under abiotic and hormone stress; it had an early response to jasmonic acid (JA) and ethephon (ET) but a late response to salicylic acid (SA) and was significantly upregulated under *Bipolaris maydis* infection. The overexpression of *ZmGLP1* in *Arabidopsis* improved the resistance to biotrophic *Pseudomonas syringae* pv. tomato DC3000 (*Pst*DC3000) and necrotrophic *Sclerotinia sclerotiorum* by inducing the expression of JA signaling-related genes. Moreover, the hydrogen peroxide (H_2_O_2_) content increased due to the overexpression of *ZmGLP1* in *Arabidopsis* after pathogen infection. Compared to the wild-type control, the H_2_O_2_ content of *ZmGLP1*-overexpressing *Arabidopsis* infected by *PstDC3000* increased significantly but was lower in transgenic plants infected with *S. sclerotiorum*. Furthermore, high-performance liquid chromatography–tandem mass (HPLC-MS/MS) spectrometry showed that the JA contents of *ZmGLP1*-overexpressing *Arabidopsis* markedly increased after pathogen infection. However, the improved resistance of *ZmGLP1*-overexpressing *Arabidopsis* pretreated with the JA biosynthetic inhibitor, sodium diethyldithiocarbamate trihydrate (DIECA), was suppressed. Based on these findings, we speculate that *ZmGLP1* plays an important role in the regulation of *Arabidopsis* resistance to biotrophic *Pst*DC3000 and necrotrophic *S. sclerotiorum*; the regulatory effects are achieved by inducing plant oxidative burst activity and activation of the JA signaling pathway.

## 1. Introduction

Maize is an important food crop, a primary source of animal feed, and an important industrial raw material used in alcohol and starch production worldwide. Maize has also been playing an increasingly important role in the global economy and agricultural production [1]. In recent years, due to the adjustment of planting structure, transformation of cultivation methods, substitution of maize varieties, and extreme climatic events, the occurrence of maize diseases has increased [2,3]. For example, the widespread cultivation of the susceptible variety Xianyu335 has led to an outbreak of northern corn leaf blight in northern China [4]. In the Huang-Huai-Hai plain of China, high temperatures and humidity have resulted in prime conditions for stalk rot and ear rot diseases [2]. These diseases have become restricting factors that prevent high and stable maize yields. Currently, the most economical and effective strategy for coping with disease stress is to explore new disease-resistant genes and cultivate new disease-resistant maize varieties.

Germin-like proteins (GLPs) are a class of disease-related proteins containing cupin domains that extensively exist in plants [5]. They are highly homologous to germin (GER) sequences and possess β-folded bucket conserved domains. GLPs are soluble proteins that belong to the cupin superfamily. To date, several GLPs have been cloned from many plant species, including *Hordeum vulgare*, *Arabidopsis thaliana*, *Triticum aestivum*, *Oryza sativa*, and *Lilium regale* Wilson [6,7,8]. The overexpression of GLP genes increases the resistance of transgenic plants to various pathogens [9,10]. For example, *AhGLPs* in peanut were significantly upregulated in response to *Aspergillus flavus* infection, indicating that *AhGLPs* are prevalent in the defense against *A. flavus* [11]. Zhang et al. identified *LrGLP1*, which confers *L. regale* Wilson resistance to *Fusarium oxysporum*, and found that *LrGLP1*-overexpressing tobacco increases disease resistance to *F. oxysporum* by enhancing the reactive oxygen species (ROS)-scavenging ability [12]. Barley *HvGLP4* and wheat *TaGLP4* significantly increase the activities of superoxide dismutase (SOD) and polyphenol oxidase (PPO) due to pathogen infection and clear ROS produced during pathogen invasion, resulting in the accumulation of hydrogen peroxide (H_2_O_2_), which activated specific H_2_O_2_-mediated defense response genes [13]. Pei et al. found that heterologous-expressing *GhABP19* in *Arabidopsis*, a member of the cotton GLP family, increases *Arabidopsis* resistance to *F. oxysporum* by activating the jasmonic acid (JA)-mediated signaling pathway [14]. The function of GLP genes in some plants has been demonstrated [12,13,14]; however, the regulatory mechanism underlying GLP genes remains unclear.

Only a few studies on GLP genes in maize have been conducted. The numbers and characteristics of *ZmGLP* genes in the maize genome and expression changes during *Setosphaeria turcica* infection have been identified and reported [8]. Few studies on the signaling pathways involved in the resistance to different trophic pathogens of GLP genes have been reported. Therefore, the isolation and study of GLP genes in maize are important for enhancing our understanding of the resistance mechanisms involved in response to diseases and are crucial for facilitating the methods and development of disease-resistant maize varieties.

In a previous study, we found that the application of *Bacillus subtilis* DZSY21 enhances maize resistance to *Bipolaris maydis* and identified differentially expressed genes that respond to pathogen infection in interactions between DZSY21 and maize [15,16]; *ZmGLP1* was identified among these genes. In this study, we isolated *ZmGLP1* from maize, analyzed bioinformatic data and tissue-specific expression patterns, induced the expression of *ZmGLP1*, and clarified the function of this gene in response to infection with different trophic pathogens, including biotrophic *Pst*DC3000 and necrotrophic *S. sclerotiorum*. Our findings will lay a foundation for clarifying the disease resistance function of *ZmGLP1* in future studies.

## 2. Results

### 2.1. Identification and Physicochemical Property Analysis of ZmGLP1

**Figure 1:**
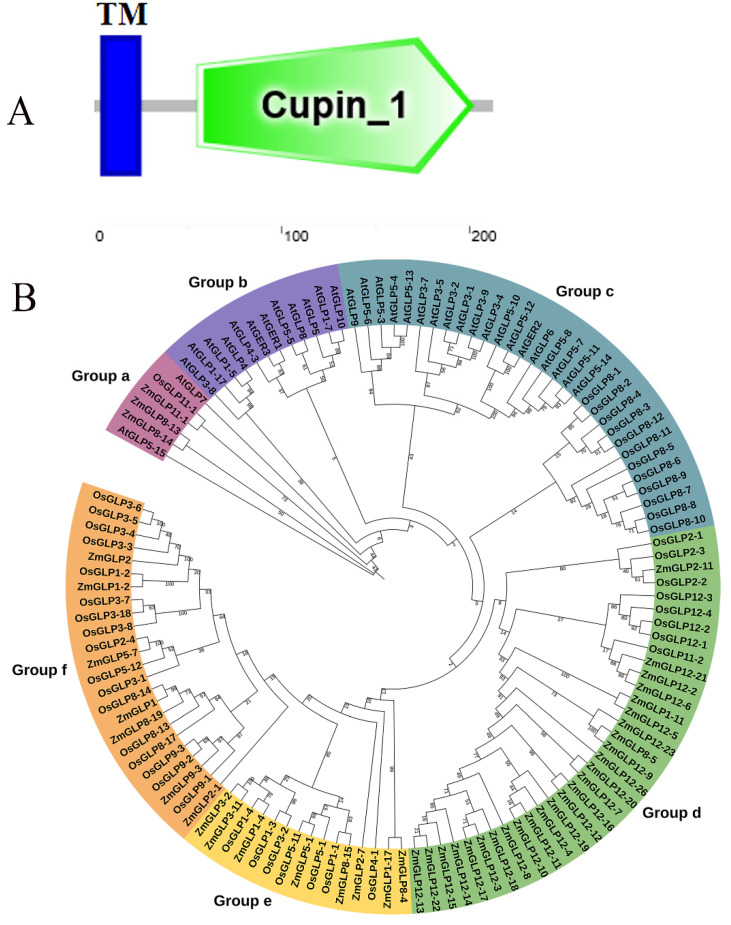
Characteristic analysis of *ZmGLP1*. (**A**) The conserved domain analysis of *ZmGLP1*. (**B**) Phylogenetic analysis of *GLP*s gene family from *At* (*Arabidopsis thaliana*), *Os* (*Oryza sativa*), and *Zm* (*Zea mays*). GLP genes are divided into 6 categories. *ZmGLP1* is marked in red. Bootstrap values of 1000 replicates are shown as percentages at the branch nodes. Bar = 0.1. Subfamilies are represented by orange, yellow, green, blue, bluish-purple, and purple branch lines.

The *ZmGLP1* (GRMZM2G064096) CDS obtained from the Phytozome database (https://plants.ensembl.org/Zea_mays/Info/Index, accessed on 20 October 2019) had a full length of 639 bp, encoded 212 amino acids, and was located on chromosome 6. The predicted isoelectric point and molecular weight of the ZmGLP1 protein were 6.01 and 54.08 kDa, respectively. It harbored a typical Cupin_1 conserved domain (Figure 1A and Figure S1), which is characteristic of the plant GLP family. Based on the 132 GLP genes identified in maize, *O. sativa*, and *Arabidopsis* [6], a phylogenetic tree was constructed using MEGA7.0 software (Figure 1B). The tree revealed that the GLP family members in maize, *O. sativa*, and *Arabidopsis* are divided into six subfamilies (Groups a–f). Moreover, maize *ZmGLP1* is clustered with subfamily b and has a close phylogenetic relationship with *OsGLP8-14* from *O. sativa* (Figure 1B; Table S2).

### 2.2. Expression of ZmGLP1 in Different Maize Tissues

**Figure 2:**
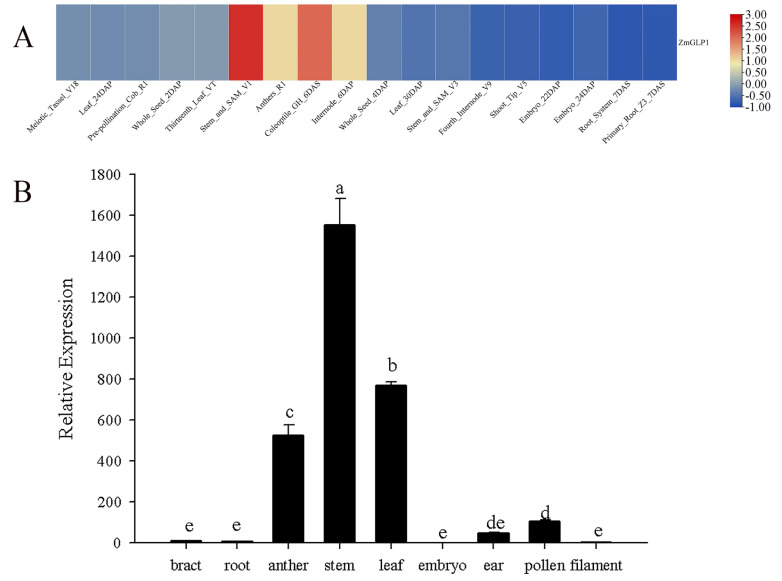
Expression analysis of *ZmGLP1* in different maize tissues. (**A**) Heat map of tissue-specific expression of *ZmGLP1* in maize. DAS = days after sowing; DAP = days after pollination. (**B**) Relative expression of *ZmGLP1* in different maize tissues. Different letters in subfigure (**B**) indicated significant difference between different samples by Duncan’s post-hoc test. Data are presented as the mean ± SD based on three biological replicates.

To clarify the tissue-specific expression pattern of the *ZmGLP1* gene in maize, publicly available corn microarray data of different tissues were applied to draw the heat map (Figure 2A). Results revealed that *ZmGLP1* is highly expressed in the stems, coleoptile, and anthers (Figure 2A). The above prediction results were verified by qRT-PCR, which found that *ZmGLP1* is expressed in all maize growth stages. The expression level of *ZmGLP1* is the highest in the stems, followed by the leaves and anthers, but lower in the roots, embryos, and filaments. These results are consistent with the chip data (Figure 2B).

### 2.3. Analysis of ZmGLP1 Expression Induced by Biotic Stress and Hormone Signals

**Figure 3:**
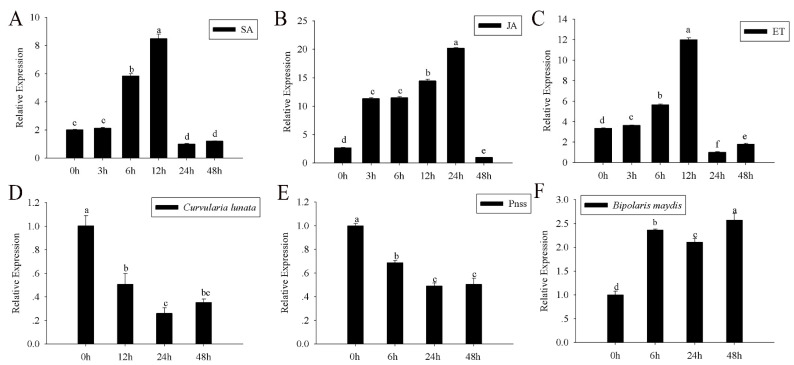
Relative expression levels of *ZmGLP1* under six different treatments over time. (**A**) Salicylic acid application, (**B**) jasmonic acid application, (**C**) ethephon application, (**D**) the conidial suspension of *Curvularia lunata*, (**E**) *Pantoea stewartii* suspensions, (**F**) the conidial suspension of *Bipolaris maydis*. *ZmActin1* and *GADPH* were used as reference genes for normalization. Different letters in subfigures (**A**–**F**) indicated significant difference between different samples by Duncan’s post-hoc test. Data are presented as the mean ± SD based on three biological replicates.

SA, JA, and ET, which mediate plant defense responses against biological stress [17], were analyzed to determine the response of *ZmGLP1* to pathogen infection. When compared to 0 h, the transcription level of *ZmGLP1* gradually increased 6–12 h after SA application, and its expression increased by 4.0 times after 12 h and then declined rapidly (Figure 3A). *ZmGLP1* gradually increased 3–24 h after JA application and reached its maximum level after 24 h, which was 10.0 times greater than that at 0 h (Figure 3B). The *ZmGLP1* mRNA transcript level increased gradually from 3 to 12 h after ET application and was 3.43 times higher at 12 h (Figure 3C). *ZmGLP1* may have different response times and expression patterns to these hormones, as *ZmGLP1* had an early response to JA and ET (3 h) but a late response to SA (6 h).

The transcription level of *ZmGLP1* decreased gradually 0–48 h after *C. lunata* and *P. stewartii* inoculation (Figure 3D,E) but slowly increased in *B. maydis*-treated plants after 0–48 h. At 48 h, the expression of *ZmGLP1* was 2.50 times greater than that at 0 h after *B. maydis* infection (Figure 3F). These results indicate that *ZmGLP1* might be involved in plant disease resistance, but its underlying mechanism requires further investigation.

### 2.4. Subcellular Localization Analysis of ZmGLP1

**Figure 4:**
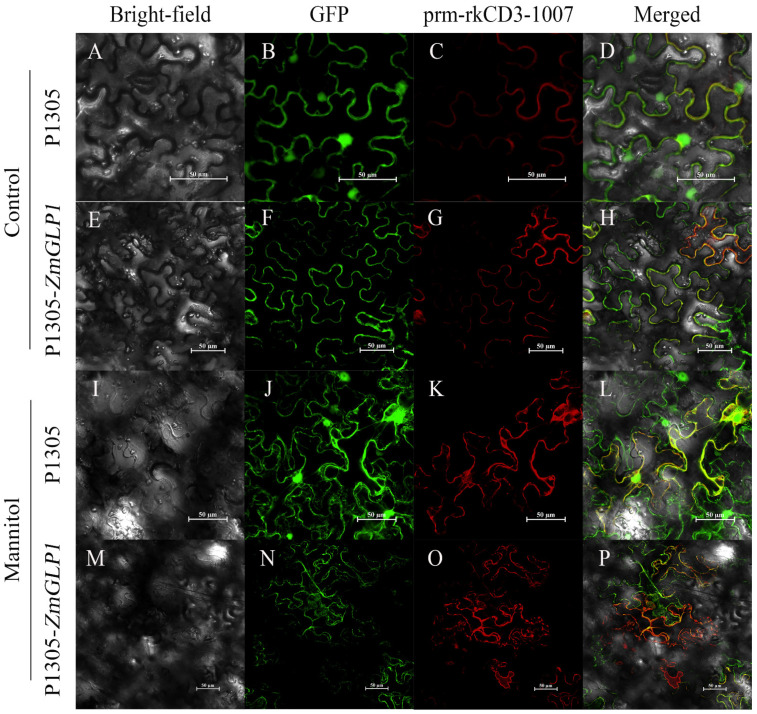
*ZmGLP1* location in the cell wall and plasma membrane. (**A**–**D**) Subcellular localization images of positive control 35S-GFP in *N. benthamiana.* (**E**–**H**) Subcellular localization of p1305-*ZmGLP1*-GFP in *N. benthamiana*. (**I**–**L**) Images of 35S-GFP epidermal cells after fugitive wall isolation. (**M**–**P**) Image of p1305-*ZmGLP1*-GFP signal in *N. benthamiana* epidermal cells after plasmolysis. Bar = 50 µm.

According to our predictions (http://psort.hgc.jp, accessed on 10 October 2020), the protein encoded by *ZmGLP1* is located in the cell wall. To verify this, p1305-*ZmGLP1*-GFP and the plasma membrane marker pm-rk CD3-1007 were co-infiltrated in *N. benthamiana*, and the fusion protein was allowed to accumulate in the cells (Figure 4A–H). To further confirm the protein location, plasmolysis was performed using *N. benthamiana* leaf epidermal cells. Results revealed that 35S-GFP and pm-rk CD3-1007 shrank during plasmolysis treatment. However, p1305-*ZmGLP1*-GFP and pm-rk CD3-1007 were isolated during the plasmolysis treatment (Figure 4I–P), indicating that *ZmGLP1*-GFP is localized in the cell wall and plasma membrane.

### 2.5. Expression of ZmGLP1 in Arabidopsis Increases Resistance to PstDC3000

**Figure 5:**
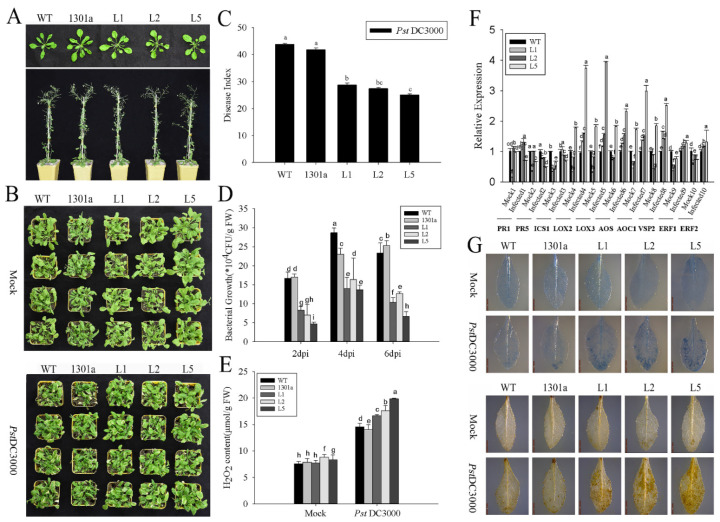
Expression of *ZmGLP1* in *Arabidopsis* increases resistance to *Pst*DC3000. (**A**) Growth status of *Arabidopsis* leaves in different groups at four and seven weeks old. (**B**) Phenotypes of the different groups treated with *Pst*DC3000 at 7 d. (**C**) The disease indexes of groups at 7 d. (**D**) The population density of bacteria of different groups. (**E**) H_2_O_2_ content of different groups at 3 d. (**F**) The expression levels of target plant genes of the salicylic acid, jasmonic acid, or ethephon pathway at 24 h. *AtActin2* and *AtTUB4* were used as reference genes for normalization. (**G**) Trypan blue staining and DAB staining in *Arabidopsis* leaves of different groups at 3 d. Different letters in subfigures (**C**–**F**) indicated significant difference between different samples by Duncan’s post-hoc test. Data are presented as the mean ± SD based on three biological replicates.

To elucidate the function of *ZmGLP1*, transgenic *Arabidopsis* plants overexpressing *ZmGLP1* were obtained (Figure S2). Three independent T_3_ homozygous lines (L1, L2, and L5) were selected to analyze the functional characteristics of *ZmGLP1* (Figure 5A). *Pst*DC3000 and *S. sclerotiorum* were used to evaluate the disease resistance of *ZmGLP1*-overexpressing *Arabidopsis* to pathogen infection.

Symptoms appeared on the leaves of different groups 3 d after foliar spraying with *Pst*DC3000 suspension (2 mL per plant). Yellow necrosis spots were observed on plant leaves after 7 d. Compared to the negative control, the leaves of transgenic *Arabidopsis* had fewer spots (Figure 5B). The disease indices of transgenic L1, L2, and L5 treated with *Pst*DC3000 after 7 d were 28.46, 27.82, and 25.40, respectively, which showed a significant downtrend when compared to wild-type (43.36) and empty plasmid-overexpressing *Arabidopsis* (40.53) (Figure 5C). These results indicate that the expression of *ZmGLP1* in *Arabidopsis* improves plant resistance to *Pst*DC3000.

The bacterial populations on plant leaves of different groups at 2, 4, and 6 d after treatment with *Pst*DC3000 were detected to clarify the relationship between the bacterial populations in transgenic *Arabidopsis* and disease resistance. Results revealed that the bacterial populations of transgenic L1, L2, and L5 were lower than those of wild-type and empty plasmid-overexpressing *Arabidopsis* at 2, 4, and 6 d. The cell count of L5 remained at 6.67 × 10^4^ CFU/g at 6 d, while those of the wild-type and empty plasmid-overexpressing *Arabidopsis* plants were 23.3 × 10^4^ and 25.3 × 10^4^ CFU/g at 6 d, respectively (Figure 5D). These results indicate that the overexpression of *ZmGLP1* in *Arabidopsis* partially enhances plant resistance to *Pst*DC3000.

As an important mechanism of plant defense responses against pathogens, plant allergic cell death limits the expansion of pathogens and leads to locally acquired resistance. In this study, different groups were stained with trypan blue 3 dpi with *Pst*DC3000, which showed that more necrotic cells accumulated near the *Pst*DC3000 infection site in the transgenic lines than in wild-type and empty plasmid-overexpressing *Arabidopsis* (Figure 5G), indicating that the overexpression of *ZmGLP1* in *Arabidopsis* induces cell death due to *Pst*DC3000 infection. This finding is consistent with a previous report [18]. Moreover, several yellowish-brown precipitates formed in the leaves of transgenic plants after DAB staining when compared to the control groups (Figure 5G), indicating that H_2_O_2_ accumulated in *ZmGLP1*-overexpressing *Arabidopsis*. Our quantitative results further showed that the H_2_O_2_ content in plants infected with *Pst*DC3000 increased significantly when compared to the negative controls (Figure 5E). The H_2_O_2_ contents in transgenic L1, L2, and L5 reached 16.68, 17.75, and 19.90 µmol/g, respectively, which were higher than those in the wild-type (14.57 µmol/g) and empty plasmid-overexpressing (14.06 µmol/g) *Arabidopsis* (Figure 5E). Results revealed that *ZmGLP1*-overexpressing *Arabidopsis* resists *Pst*DC3000 infection by inducing more cell death and accumulating H_2_O_2_.

To study the signaling pathways that *ZmGLP1* participates in and thereby enhances resistance of transgenic *Arabidopsis* to *Pst*DC3000, the expression levels of target plant genes in the SA, JA, or ET signaling pathways were analyzed [19]. At 24 h after spraying with *Pst*DC3000, *LOX2*, *LOX3, AOC1*, *AOS*, and *VSP2* were strongly induced in transgenic lines, and the expression levels of these genes in L5 were 3.75, 3.94, 2.98, 2.32, and 2.52 times higher than those in wild-type plants, respectively (Figure 5F). *ERF1* and *ERF2* were slightly induced, while *PR1*, *PR5*, and *ICS1* were not induced in transgenic *Arabidopsis* (Figure 5F). Results revealed that *LOX2*, *LOX3*, and *AOC1* were simultaneously highly expressed in *ZmGLP1*-overexpressing lines after *Pst*DC3000 infection, indicating that *ZmGLP1* may participate in plant defense mechanisms against biotrophic *Pst*DC3000 by activating the JA-dependent signaling pathway.

### 2.6. ZmGLP1 Confers Resistance to S. sclerotiorum in Arabidopsis

**Figure 6:**
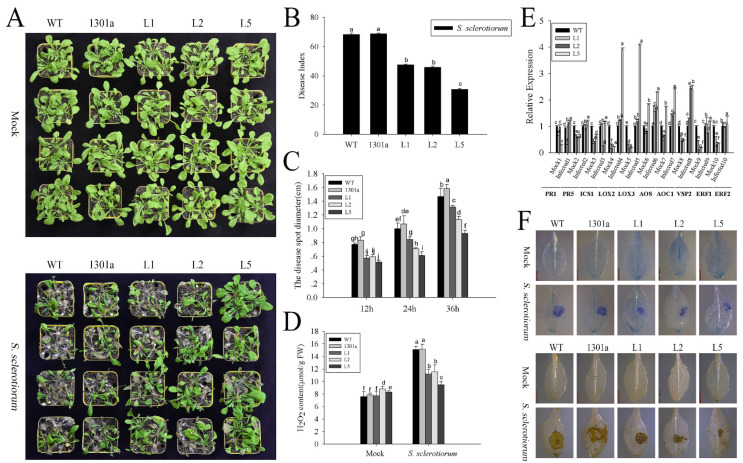
*ZmGLP1* increases resistance to *Sclerotinia sclerotiorum* in *Arabidopsis*. (**A**) Phenotypes of different groups before and after being treated with *Sclerotinia sclerotiorum* at 3 d. (**B**) The disease indexes of different groups at 3 d. (**C**) The disease spot diameter of the *Arabidopsis* leaves in different groups treated with *Sclerotinia sclerotiorum* at 12, 24, and 36 h. (**D**) H_2_O_2_ content in *Arabidopsis* leaves of different groups at 3 d. (**E**) The expression levels of target plant genes of the salicylic acid, jasmonic acid, or ethephon pathway at 6 h. *AtActin2* and *AtTUB4* were used as reference genes for normalization. (**F**) Trypan blue staining and DAB staining of different groups of leaves at 3 d. Different letters in subfigures (**B**–**E**) indicated significant difference between different samples by Duncan’s post-hoc test. Data are presented as the mean ± SD based on three biological replicates.

After clarifying disease resistance in *ZmGLP1*-overexpressing plants to *Pst*DC3000, the function of *ZmGLP1* in transgenic plants against pathogenic necrotrophic *S. sclerotiorum* was analyzed. At 12 h post infection with *S. sclerotiorum*, necrotic lesions appeared near the inoculation sites. The lesion diameters in transgenic plants were significantly smaller than those in wild-type and empty plasmid-overexpressing plants at 12, 24, and 36 h. The lesion diameter in L5 was 0.93 cm at 36 h, while that of the wild-type and empty plasmid-overexpressing *Arabidopsis* reached 1.46 and 1.58 cm at 36 h, respectively (Figure 6C). The disease indices of L1 (47.24), L2 (45.83), and L5 (30.21) were significantly lower than those of the wild-type (68.78) and empty plasmid-overexpressing *Arabidopsis* (69.35) at 3 dpi with *S. sclerotiorum* (Figure 6B). Transgenic L5 especially showed stronger disease resistance to *S. sclerotiorum* (Figure 6A,C). These results indicate that *ZmGLP1*, which is overexpressed in *Arabidopsis*, partially increases resistance to *S. sclerotiorum*.

Subsequently, the groups were inoculated with *S. sclerotiorum* for 3 d, then stained with trypan blue and DAB. We found fewer necrotic cells around the lesions in the transgenic lines compared to the wild-type and empty plasmid-overexpressing *Arabidopsis*. The lesions in *ZmGLP1*-overexpressing lines were smaller than those in other groups (Figure 6F). H_2_O_2_ accumulated on the surface of spots in different groups treated with *S. sclerotiorum* (Figure 6F). The H_2_O_2_ contents of the wild-type, empty plasmid-overexpressing, and transgenic L1, L2, and L5 plants increased significantly, reaching 15.10, 15.16, 11.20, 11.55, and 9.48 µmol/g, respectively, when compared to the negative controls (Figure 6D). However, the H_2_O_2_ contents of transgenic plants were lower than those of the wild-type and empty plasmid-overexpressing *Arabidopsis* (Figure 6D).

Finally, the key genes in transgenic plants were analyzed to investigate the disease resistance signaling pathway that *ZmGLP1* participates in and thereby enhances resistance to *S. sclerotiorum.* At 6 h after infection with *S. sclerotiorum*, the target genes in the JA signaling pathway in transgenic L1, L2, and L5 were upregulated. *LOX2*, *LOX3, AOC1*, *AOS*, and *VSP2* in transgenic L5 were strongly induced and were 3.93, 4.08, 2.41, 2.30, and 2.41 times higher than those in wild-type plants, respectively (Figure 6E). *ERF1* and *ERF2* in L5 were slightly induced and were 1.66 and 1.80 times greater than those in wild-type plants (Figure 6E). These results indicate that *ZmGLP1* might participate in plant defense mechanisms against pathogenic fungi by activating the JA-dependent signaling pathway.

### 2.7. ZmGLP1-Overexpressing Arabidopsis Exhibits Resistance to PstDC3000 and S. sclerotiorum through the JA-Mediated Signaling Pathway

**Figure 7:**
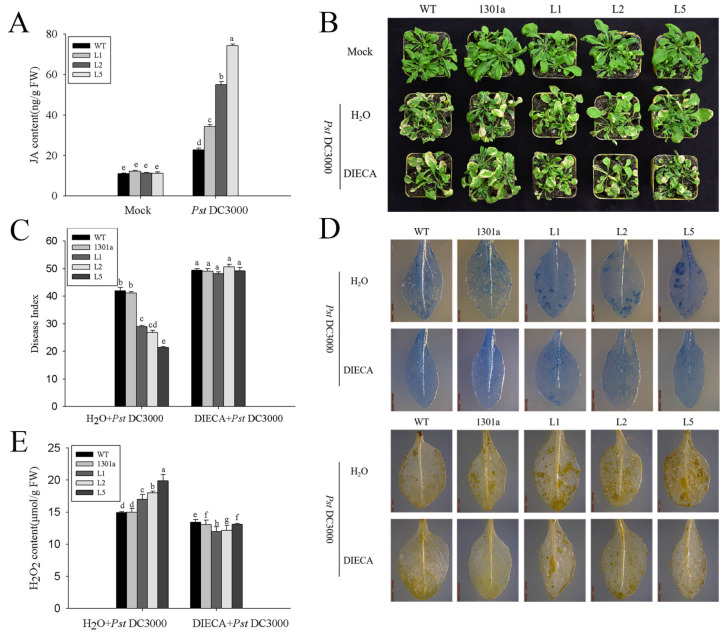
Overexpression of *ZmGLP1* in *Arabidopsis* enhances resistance to *Pst*DC3000 through the jasmonic-acid-mediated signaling pathway. (**A**) Jasmonic acid contents in the different groups after *Pst*DC3000 infection. (**B**) Phenotypes of the different groups at 7 d post *Pst*DC3000 infection, which were pretreated with DIECA for 24 h. (**C**) Disease indexes of the different groups. (**D**) Trypan blue staining and DAB staining of the different groups. (**E**) Detection of H_2_O_2_ content of *Arabidopsis* leaves at 3 d post *Pst*DC3000 infection in different groups shown in (**B**). Different letters in subfigures (**A**,**C**,**E**) indicated significant difference between different samples by Duncan’s post-hoc test.Data are presented as the mean ± SD based on three biological replicates.

**Figure 8:**
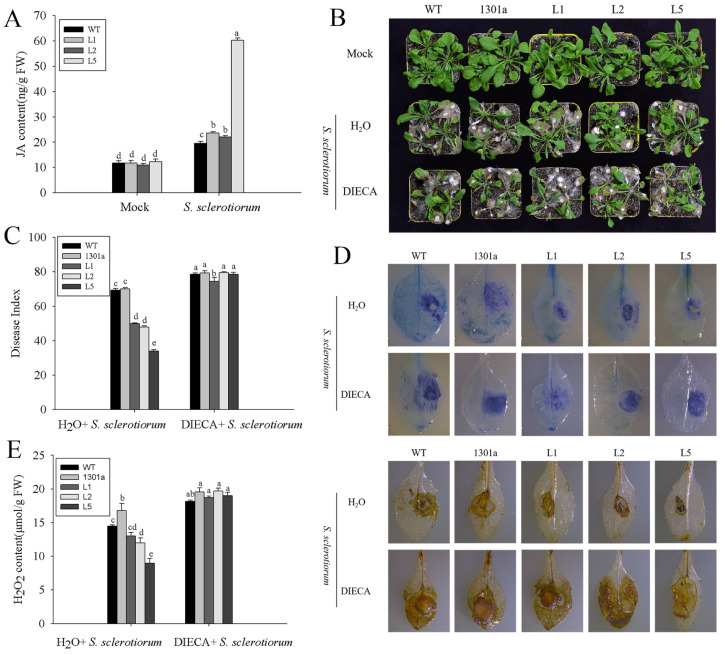
*ZmGLP1* transgenic *Arabidopsis* enhances resistance to *Sclerotinia sclerotiorum* through the jasmonic-acid-mediated signaling pathway. (**A**) Jasmonic acid contents in the different groups at 6 h post *Sclerotinia sclerotiorum* infection. (**B**) Phenotypes of the different groups at 3 d post *Sclerotinia sclerotiorum*, which were pretreated with DIECA for 24 h. (**C**) Disease indexes of the different groups. (**D**) Trypan blue staining and DAB staining of the different groups. (**E**) Detection of H_2_O_2_ content of *Arabidopsis* leaves at 3 d in different groups. Different letters in subfigures (**A**,**C**,**E**) indicated significant difference between different samples by Duncan’s post-hoc test. Data are presented as the mean ± SD based on three biological replicates.

Preliminary results indicated that *ZmGLP1* might participate in plant defense mechanisms against *Pst*DC3000 and *S. sclerotiorum* through the JA signaling pathway. However, the dynamic content changes of endogenous JA in transgenic *Arabidopsis* before and after pathogen infection have yet to be determined. Therefore, the JA contents in *ZmGLP1*-overexpressing *Arabidopsis* were analyzed by HPLC-MS/MS. The retention time of the peaks of the JA extracts was consistent with standard JA, appearing at 8.09 ± 0.02 s (Figures S3 and S4). The JA contents of transgenic and wild-type plants did not exhibit apparent differences before *Pst*DC3000 or *S. sclerotiorum* infection; however, the JA contents of transgenic L1, L2, and L5 accumulated rapidly after pathogen infection and reached 33.18, 52.59, and 71.71 μg/g FW at 24 h after *Pst*DC3000 infection, respectively, which were significantly higher than those of wild-type plants (21.84 μg/g FW) (Figure 7A). Furthermore, the JA contents in L1, L2, and L5 infected with *S. sclerotiorum* were 22.23, 20.53, and 57.71 μg/g FW, respectively, which were also significantly higher than those of wild-type plants (18.59 μg/g FW) (Figure 8A). These results show that the rapid accumulation of endogenous JA in transgenic *Arabidopsis* enhances disease resistance to *Pst*DC3000 and *S. sclerotiorum* as well as indicates that the overexpression of *ZmGLP1* in *Arabidopsis* may activate the JA signaling pathway to enhance pathogen resistance.

DIECA is a JA biosynthesis inhibitor that effectively controls JA biosynthesis in plants. To confirm that *ZmGLP1* enhances *Arabidopsis* resistance to *Pst*DC3000 and *S. sclerotiorum* through the JA signaling pathway, different groups were pretreated with 200 μmol/L DIECA for 24 h. Then, plants were infected with *Pst*DC3000 or *S. sclerotiorum*; disease indices were estimated at 7 and 3 dpi with *Pst*DC3000 and *S. sclerotiorum*, respectively. As expected, *ZmGLP1*-overexpressing lines L1, L2, and L5 and empty plasmid-overexpressing and wild-type plants pretreated with DIECA exhibited more severe symptoms after inoculation with *Pst*DC3000 (Figure 7B); the disease indices were 48.16, 50.68, 49.16, 49.05, and 49.40, respectively (Figure 7C), while the negative groups were 28.95, 26.77, 21.43, 41.12, and 41.96, respectively (Figure 7C). The disease indices of transgenic lines L1, L2, and L5 and wild-type and empty plasmid-overexpressing *Arabidopsis* after *S. sclerotiorum* infection were 74.41, 79.47, 78.62, 78.47, and 79.28, respectively (Figure 8B, C), while the disease indices of the negative groups were 49.92, 47.81, 33.86, 69.32, and 70.07, respectively (Figure 8C). These results indicate that the application of DIECA reduces disease resistance to *Pst*DC3000 and *S. sclerotiorum* in transgenic plants.

Trypan blue was used to detect necrotic cells in plant leaves infected with *Pst*DC3000 and *S. sclerotiorum*. Results revealed that hypersensitive reactions were inhibited in DIECA-pretreated *Arabidopsis* lines infected with *Pst*DC3000, which had fewer necrotic cells around the infection site when compared to the negative controls (Figure 7D). Fewer yellowish-brown precipitates were observed in DIECA-pretreated *Arabidopsis* leaves after 3 dpi with *Pst*DC3000 (Figure 7D). The H_2_O_2_ contents in the DIECA-pretreated groups significantly decreased when compared to the negative controls (Figure 7E).

Trypan blue and DAB staining were performed so we could observe the number of necrotic cells and the active site of peroxidase in DIECA-pretreated plants after 3 dpi with *S. sclerotiorum*. Results revealed that more necrotic cells and yellowish-brown precipitates aggregated near the lesions on DIECA-pretreated plant leaves when compared to the negative controls (Figure 8D). The H_2_O_2_ content of the DIECA-pretreated lines significantly increased when compared to the negative controls, exhibiting the opposite trend to DIECA-pretreated groups inoculated with *Pst*DC3000 (Figure 8E). These results indicate that DIECA application reduces disease resistance in *ZmGLP1*-overexpressing plants to *Pst*DC3000 and *S. sclerotiorum*, further indicating that the overexpression of *ZmGLP1* in *Arabidopsis* enhances resistance to biotrophic *Pst*DC3000 and necrotrophic *S. sclerotiorum* by activating the JA signaling pathway.

## 3. Materials and Methods

### 3.1. Bioinformatics Analysis of ZmGLP1

The *ZmGLP1* coding sequence (CDS) and protein sequence were downloaded from the Phytozome database (http://phytozome.jgi.doe.gov/pz/portal.html, accessed on 20 October 2019). A conserved motif in the *ZmGLP1* protein was detected by SMART (http://smart.embl-heidelberg.de/, accessed on 20 October 2019). Then, using MEGA v7.0 software, the obtained sequences were clustered by the neighbor-joining (NJ) method, and the constructed phylogenetic tree was optimized using the ITOL website (https://itol.embl.de/, accessed on 20 January 2021). The data required for drawing a heat map of the tissue-specific expression of *ZmGLP1* were obtained from the Corn database (http://www.plexdb.org/plex.php?database=Corn, accessed on 20 January 2021); a heat map was drawn following previously described methods [20,21].

### 3.2. Vector Construction and Arabidopsis Transformation

*ZmGLP1* was cloned from maize leaves by PCR using specific primers. The mRNA was cloned by RT-PCR (forward: ATG GCC AAA ATG GTG TTG; reverse: ACC GCT GCC GCC GAG CAC AC). The fragment was inserted (digested with BamHI and PstI) into the vector pCAMBIA1301 to generate p35S:*ZmGLP1*, and the designed primer sequences were as follows: *ZmGLP1*-BamHI: GGG GAT CCA TGG CCA AAA TGG TGT TGC TCT GC; *ZmGLP1*-PstI: GCC TGC AGA CCG CTG CCG CCG AGC A (Figure S5). Then, plasmid correctness was confirmed by enzyme digestion analysis and sequencing. *ZmGLP1* transgenic *Arabidopsis* was generated by *Agrobacterium*-mediated genetic transformation [21]. Transgenic T3 homozygous *Arabidopsis* was used in the follow-up experiments.

### 3.3. Pathogen Cultivation

*Pseudomonas syringae* pv. *tomato* DC3000 (*Pst*DC3000) was grown at 28 °C for 24 h in a beef protein liquid medium. *S. sclerotiorum* was incubated on PDA solid medium at 22 °C for 2 d under a 12/12 h light/dark photoperiod.

### 3.4. Plant Materials and Treatments

Maize (B73) plants and *Nicotiana benthamiana* were grown at 25 °C under a 16/8 h light/dark photoperiod and 60–70% relative humidity. *Arabidopsis* was grown at 23 °C under a 16/8 h light/dark photoperiod.

Analyses of *ZmGLP1* expression induced by biotic stress and hormones and the tissue-specific expression of *ZmGLP1* were conducted following previously described methods [21]. Plants at the three-leaf stage were treated with salicylic acid (SA) (1 mM), jasmonic acid (JA) (50 μM), or ethephon (ET) (1 mM) and infected with *Curvularia lunata* (1 × 10^5^ CFU/mL), *Bipolaris maydis* (1 × 10^5^ CFU/mL), or *Pantoea stewartii* (1 × 10^6^ CFU/mL). At 0, 3, 6, 12, 24, and 48 h after pathogen and hormone infection, maize leaves were collected for RNA extraction. Maize tissues at different growth stages were collected to extract RNA for the analysis of the tissue-specific expression of *ZmGLP1*. Each group consisted of three replicates (three maize plants per replicate).

Four-week-old *Arabidopsis* sprayed with *Pst*DC3000 suspension (OD_600_ = 0.4) and plants treated with 10 mM MgCl_2_·6H_2_O were used as the negative controls. Disease severity was recorded 7 days post infection (dpi) with a scoring range of 0–4 [22]. Grades of 0, 1, 2, 3, and 4 indicated diseased leaf areas of 0%, 1–25%, 26–50%, 51–75%, and 76–100%, respectively. Three leaves were picked from each wild and transgenic *Arabidopsis* plant at the age of 4 weeks, and each leaf was inoculated on a quarter of an *S. sclerotiorum* dish (Φ = 6 mm); plants without *S. sclerotiorum* inoculation were used as the negative controls. The size of the lesion diameter was measured at 12, 24, and 36 h post infection, and the severity of the disease was recorded at 3 d with a scoring range of 0–4. Grades of 0, 1, 2, 3, and 4 indicated diseased leaf areas of 0%, 0–10%, 11–30%, 31–50%, and 50–100%, respectively [23]. There were 30 plants in a single treatment, and the plants within each treatment were divided into three replicates. The disease index was calculated to evaluate the disease resistance of plants [15]. *Arabidopsis* leaves were collected for RNA extraction at 24 h after infection with *Pst*DC3000 and 6 h with *S. sclerotiorum*.

Bacterial numbers within the mesophyll tissues were estimated using the serial dilution coating method on different days after *Pst*DC3000 infection [24]. Each group consisted of three replicates (three *Arabidopsis* plants per replicate).

To confirm that the overexpression of *ZmGLP1* in *Arabidopsis* enhances pathogen resistance through the JA signaling pathway, four-week-old wild-type and transgenic *Arabidopsis* were treated with 200 μmol/L sodium diethyldithiocarbamate trihydrate (DIECA) containing 0.02% Tween 20. After 24 h, the plants were inoculated with *Pst*DC3000 suspension and *S. sclerotiorum*. Plants pretreated with sterile distilled water containing 0.02% Tween 20 were used as the negative controls. A total of 30 plants in each treatment were divided into three replicates per treatment.

### 3.5. Quantitative PCR

Plant leaves harvested at different time points were ground in liquid nitrogen for RNA extraction with TRIzol reagent. The RNA quality was evaluated based on the concentration using electrophoresis strips. qRT-PCR was conducted using AceQ qPCR SYBR Green Master Mix (Vazyme Biotech Co., Ltd., Nanjing, China). Each PCR tube contained 10 μL AceQ qPCR SYBR Green Master Mix, 25 ng cDNA, and 1 μL of each primer [25] (Table S1). The thermal cycling conditions were as follows: 30 s at 94 °C, 40 cycles for 5 s at 94 °C, and 15 s at 55 °C. The data were processed following previously described methods [26].

### 3.6. Subcellular Localization Assay of ZmGLP1

The p1305-*ZmGLP1*-GFP vector was constructed (Figure S6). Through sequence analysis, we inserted the target fragment, which removed the terminator, into the front of the GFP tag by double digestion sites (XbaI and BamHI). The designed primer sequences were as follows: *ZmGLP1*-XbaI: GCT CTA GAA TGG CCA AAA TGG TGT TGC TCT GC; *ZmGLP1*-BamHI: ATG GAT CCA CCG CTG CCG CCG AGC A. Then, plasmid correctness was confirmed by enzyme digestion analysis and sequencing. The vector was transformed into *Agrobacterium tumefaciens* strain GV3101 for *Agrobacterium*-mediated transient expression. Four-week-old *N. benthamiana* was selected for infiltration treatment. The p1305-*ZmGLP1*-GFP or 35S-GFP vectors and a plasma membrane marker (pm-rk188 CD3-1007) were co-infiltrated in *N. benthamiana* leaves for 3 d [27]. Subsequently, *N. benthamiana* was subjected to plasmolysis, and the infiltrated leaves were incubated in 1 mM mannitol for 30 min. The fluorescence of the samples was observed under a Nikon AX confocal microscope (Nikon, Tokyo, Japan).

### 3.7. DAB Staining

To detect the degree of H_2_O_2_ accumulation in *ZmGLP1*-overexpressing *Arabidopsis* leaves at 3 dpi, DAB staining was applied to the leaves. Under the catalysis of peroxidase, H_2_O_2_ rapidly reacts with DAB to form brown compounds, thereby revealing brown spots on plant leaves. Therefore, the aggregation of H_2_O_2_ can be determined by observing whether brown spots are produced on the leaves. The size and color of the spots produced by H_2_O_2_ accumulation are often used to qualitatively analyze the accumulation of H_2_O_2_ in plants under stress.

At 3 dpi with *Pst*DC3000 and *S. sclerotiorum*, *Arabidopsis* leaves were collected and completely immersed in a Petri dish filled with 10 mM DAB staining solution, soaked in a vacuum for 5 min, wrapped with tin foil, and gently placed on a horizontal shaker at 80 rpm for 4–5 h. Then, the DAB staining solution was changed to a bleach solution and transferred to a water bath at 90–95 °C for 15 min to rinse off the chlorophyll, thereby leaving a brown precipitate from the DAB and H_2_O_2_ reaction. Samples were left in the new bleach solution for 30 min and then placed for 4 d at 4 °C [28]. *Arabidopsis* leaves treated with 10 mM MgCl_2_·6H_2_O and uninoculated leaves were used as the negative controls for the *Pst*DC3000 and *S. sclerotiorum* treatments, respectively.

### 3.8. Detection of H_2_O_2_

Wild-type and transgenic *Arabidopsis* leaves were harvested at 3 dpi and stored at −80 °C for detection of the H_2_O_2_ content using a Keming kit (Suzhou Keming Biotechnology Co., Ltd., Suzhou, China). Based on whether an orange complex was formed by the H_2_O_2_ and titanium ions in the acidic media, titanium sulfate spectrophotometry was used to determine the H_2_O_2_ concentrations in plants treated with the pathogens. *Arabidopsis* leaves treated with 10 mM MgCl_2_·6H_2_O and uninoculated leaves were used as the negative controls for the *Pst*DC3000 and *S. sclerotiorum* treatments, respectively.

### 3.9. Trypan Blue Staining

Trypan blue staining was performed following previously described methods [28]. Briefly, leaves infected with pathogens for 3 d were transferred to a solution containing 2.5 mg/mL trypan blue, vacuum infiltrated for 10 min, boiled for 2 min, and kept on a horizontal shaker for 12 h. Finally, the solution was replaced with a 2.5 g/mL chloral hydrate solution for decolorization until colorless. Transgenic and wild-type leaves treated with 10 mM MgCl_2_·6H_2_O or uninoculated leaves were used as the negative controls for the *Pst*DC3000 and *S. sclerotiorum* treatments, respectively.

### 3.10. Detection of JA in ZmGLP1-Overexpressing Arabidopsis

The JA contents of wild-type and transgenic *Arabidopsis* before and after 24 h infection with *Pst*DC3000 and 6 h with *S. sclerotiorum* were monitored using a high-performance liquid chromatography–tandem mass (HPLC-MS/MS) spectrometer consisting of an Agilent 1290 HPLC system coupled to an MS/MS spectrometer (Applied Biosystems 6500 Quadrupole Trap) (Agilent, San Francisco, USA), which was operated in multiple-reaction monitoring mode (Nanjing Convinced-Test Technology Co., Ltd., Nanjing, China). The JA content was extracted using the isopropanol–water–hydrochloric acid extraction method. Samples were detected by gradient elution using 0.1% formic acid methanol (A) and 0.1% acetic acid in water (B) as the mobile phases. Each group consisted of three replicates (three *Arabidopsis* plants per replicate).

### 3.11. Statistical Analysis

DPS software was used for the statistical data analyses. Statistical differences between different samples using a *P* < 0.05 threshold were assessed by one-way ANOVA followed by Duncan’s post hoc test.

## 4. Discussion

A candidate disease-resistant gene, *ZmGLP1*, was investigated in this study. *ZmGLP1* encodes a GLP and has the highest sequence similarity with *OsGLP8-14* in rice, which encodes a cell-wall-related protein with SOD activity and plays an important role in plant disease resistance [29,30]. GLP genes are widely expressed in multiple organs of different plants, showing tissue specificity and functional diversity at different developmental stages [11,31]. Our results are consistent with previous reports, which found that *ZmGLP1* is expressed in different maize tissues and mainly expressed in the stems, leaves, and anthers, suggesting that *ZmGLP1* may play an important role in maize growth and development. Additionally, we used subcellular localization analysis and plasmolysis experiments combined with network positioning to predict the localization of *ZmGLP1* in the cell wall and plasma membrane. In future studies, we intend to conduct protein fractionation coupled with Western blot analysis to confirm *ZmGLP1* subcellular localization in subsequent tests.

Plant GLPs play key roles in plant responses to pathogenic microbial infection and external stress [5]. Thus far, 32, 43, 57, and 21 GLP genes have been identified in *Arabidopsis*, *O. sativa*, *Z. mays,* and *H. vulgare*, respectively [6,7,8]. Here, we identified certain feature genes in *ZmGLP1*-overexpressing *Arabidopsis* which are involved in the SA, JA, and ET signaling pathways [19] to clarify the function of *ZmGLP1* in conferring plant disease resistance. The results demonstrated that the expression of key genes in *ZmGLP1*-overexpressing *Arabidopsis*, namely, *LOX2*, *LOX3*, *AOC1*, and *AOS*, is strongly induced after *Pst*DC3000 and *S. sclerotiorum* infection. *LOX2*, *LOX3*, *AOC1*, and *AOS* are biosynthetic marker genes of the JA signaling pathway. Thus, we speculated that *ZmGLP1*-overexpressing *Arabidopsis* might enhance its resistance by activating the JA signaling pathway in response to pathogen infection. Other studies showed that the JA contents in *ZmGLP1*-overexpressing plants rapidly accumulated 24 h after infection with *Pst*DC3000 and 6 h with *S. sclerotiorum*. However, after the application of the JA biosynthetic inhibitor DIECA, *ZmGLP1* transgenic plants did not exhibit pathogen resistance. Our findings showed that the overexpression of *ZmGLP1* promoted the accumulation of endogenous JA and conferred plant resistance to *Pst*DC3000 and *S. sclerotiorum*, suggesting that *ZmGLP1* may be involved in disease resistance, depend on the JA signaling pathway, and be conversely regulated by JA. In future studies, we will utilize the mutation coi1, which is the receptor of JA, to confirm the function of JA signaling in *ZmGLP1*-mediated defense responses.

It is well known that JA negatively regulates *Arabidopsis* defense responses to *Pst*DC3000 [32,33]. In recent years, some studies have reported interesting results that contradict previous reports. De Torres Zabala et al. revealed that JAZ5 and JAZ10 function cooperatively to attenuate phytotoxicity mediated by COR and moderately restrict *Pst*DC3000 growth; the JA in *Arabidopsis* leaves did not accumulate until late in the infection process challenged with COR-deficient *P. syringae* or in the more resistant JA receptor mutant *coi1*. [34]. The ectopic expression of *MdHIR4* in *Arabidopsis* and *N. benthamiana* enhances resistance to *Pst*DC3000. Furthermore, the interaction between MdHIR4 and AtJAZs proteins (AtJAZ3, AtJAZ4, and AtJAZ9) implies that *MdHIR4* mediates biotic stress through the JA signaling pathway [35]. Additionally, the overexpression of *PevD1* in *Arabidopsis*, which is isolated from *Verticillium dahlia*, triggered *Arabidopsis* resistance to *Pst*DC3000 by affecting the JA signaling pathway, three critical regulators of JA biosynthesis were upregulated, and the JA levels in transgenic *Arabidopsis* slightly increased [36]. In our study, we found that the ectopic expression of *ZmGLP1* plays a significant role in the regulation of resistance to biotrophic *Pst*DC3000 in *Arabidopsis* due to its ability to activate the JA pathway, which corroborates previous studies.

When plants interact with pathogenic microorganisms, the expression levels of GLP genes in plants are significantly upregulated, which induces an “oxidative burst” response. ROS burst is the main immune response in plant resistance to pathogen invasion, which directly inhibits pathogen growth and limits pathogen invasion by strengthening and thickening the cell wall. Some studies have uncovered the function of GLPs in inducing the “oxidative burst” response [37]. During the process of pathogen infection, *HvGLP4* in *H.vulgare*, *TaGLP4* in *T. aestivum*, and *OsRGLP2* and *OsGLP1* in *O. sativa* induce the burst of ROS, resulting in the accumulation of H_2_O_2_, which thereby activates genes involved in H_2_O_2_-mediated defense responses and improves disease resistance [13,28,38]. Therefore, the H_2_O_2_ contents in *ZmGLP1*-overexpressing *Arabidopsis* after pathogen infection were detected in this study. We found that the H_2_O_2_ contents in *ZmGLP1*-overexpressing *Arabidopsis* increased significantly after *PstDC3000* and *S. sclerotiorum* infection when compared to the negative controls. Interestingly, the H_2_O_2_ contents in transgenic plants were significantly higher than those in the wild-type plants infected with *PstDC3000* (Figure 5E), while the H_2_O_2_ contents in transgenic plants were lower than those in the control groups after *S. sclerotiorum* infection (Figure 6D). Changes in the H_2_O_2_ contents in transgenic plants, which responded to *Pst*DC3000 and *S. sclerotiorum* infection, exhibited the opposite trend. We speculated that *ZmGLP1*-overexpressing *Arabidopsis* might elicit different ROS responses to different types of pathogen infection. When *Pst*DC3000, a biotrophic pathogen, infects *Arabidopsis*, the intracellular receptor kinase, RIPK, recognizes the lipopolysaccharide of *Pst*DC3000 and phosphorylated NADPH oxidase RBOHD to induce oxidative burst, which initiates plant defense signal transmission, strengthens the cell wall, causes an allergic reaction, and leads to programmed cell death, thereby inhibiting the infection of pathogenic bacteria [39,40]. However, ROS play the opposite role in the infection of necrotrophic pathogens [41,42]. As a necrotrophic pathogen, *S. sclerotiorum* kills host cells by secreting toxins or cytolytic enzymes and invades the host. It uses host ROS to enhance its pathogenicity, thereby reducing the level of ROS in the host, which may prevent cell death and inhibit the growth of *S. sclerotiorum* [43]. These results support previous findings.

In summary, our data reveal that *ZmGLP1* overexpression confers disease resistance to biotrophic *Pst*DC3000 and necrotrophic *S. sclerotiorum* by inducing oxidative burst activity and promoting JA accumulation in plants, which preliminarily explicates the disease resistance signaling pathway induced by *ZmGLP1*. Future studies should aim to confirm the function of the JA signaling pathway in *ZmGLP1*-mediated defense responses by utilizing the *Arabidopsis* mutation *coi1* to enhance our understanding of the regulatory mechanism underlying the JA signaling pathway induced by *ZmGLP1* overexpression as well as identify the interacting proteins of *ZmGLP1*.

## Data Availability

Not applicable.

## Supplementary Materials

The following supporting information can be downloaded at: https://www.mdpi.com/article/10.3390/ijms232214316/s1. Figure S1: Molecular characterization of ZmGLP1; Figure S2: De-tection of ZmGLP1 transgenic Arabidopsis thaliana; Figure S3: Chromatogram of JA extracted from Arabidopsis; Figure S4: Chromatogram of JA extracted from Arabidopsis; Figure S5: Construction of the p1301-ZmGLP1 vector; Figure S6: Construction of the p1305-ZmGLP1-GFP vector; Table S1: GLP gene family in Oryza sativa and Arabidopsis and Maize; Table S2: Primers of qRT-PCR used in this study.

## References

[1] Lu X., Liu J., Ren W., Yang Q., Chai Z., Chen R., Wang L., Zhao J., Lang Z., Wang H. (2018). Gene-indexed mutations in maize. Mol. Plant.

[2] Duan C., Song F., Sun S., Guo C., Zhu Z., Wang X. (2019). Characterization and molecular mapping of two novel genes resistant to pythium stalk rot in maize. Phytopathology.

[3] Sun X., Qi X., Wang W., Liu X., Zhao H., Wu C., Chang X., Zhang M., Chen H., Gong G. (2020). Etiology and symptoms of maize leaf spot caused by Bipolaris spp. in Sichuan, 2020, China. Pathogens.

[4] Pu Z. (2013). Dynamic analysis on the disease progression of maize leaf blight between Xianyu335 and improved Xianyu335 in Western Region of Heilongjiang Province. J. Maize Sci..

[5] Ilyas M., Naqvi S.M.S., Mahmood T. (2016). In silico analysis of transcription factor binding sites in promoters of germin-like protein genes in rice. Arch. Biol. Sci..

[6] Li L., Xu X., Chen C., Shen Z. (2016). Genome-wide characterization and expression analysis of the germin-like protein family in rice and *Arabidopsis*. Int. J. Mol. Sci..

[7] Zimmermann G., Baumlein H., Mock H.P., Himmelbach A., Schweizer P. (2006). The multigene family encoding germin-like proteins of barley. Regulation and function in Basal host resistance. Plant Physiol..

[8] Liu Y.J., Jia M.X., Zhou H., Zhang L., Liu C., Si H.L., Liu Y.Q., Gu S.Q., Gong X.D., Dong J.G. (2021). Genome-wide Identification of GLP Gene Family in Maize (Zea mays) and It’s Expression Analysis When Maize is Exposed to *Setosphaeria turcica*. Chin. J. Agric. Biotechnol..

[9] Breen J., Bellgard M. (2010). Germin-like proteins (GLPs) in cereal genomes: Gene clustering and dynamic roles in plant defence. Funct. Integr. Genom..

[10] Majeed N., Javaid B., Deeba F., Naqvi S., Douches D. (2018). Enhanced Fusarium oxysporum f. sp. tuberosi Resistance in Transgenic Potato Expressing a Rice GLP Superoxide Dismutase Gene. Am. J. Potato Res..

[11] Wang T., Chen X., Zhu F., Li H., Li L., Yang Q. (2013). Characterization of peanut gennin-like proteins, AhGLPs inplant development and defense. PLoS ONE.

[12] Zhang N., Guan R., Yang Y., Bai Z., Ge F., Liu D. (2017). Isolation and characterization of a Fusarium oxysporum-resistant gene LrGLP1 from Lilium regale Wilson. In Vitro Cell Dev. Biol.-Plant.

[13] Christensen A.B., Thordal-Christensen H., Zimmermann G., Gjetting T., Lyngkjær M.F., Dudler R., Schweizer P. (2004). The germinlike protein GLP4 exhibits superoxide dismutase activity and is an important component of quantitative resistance in wheat and barley. Mol. Plant-Microbe Interact..

[14] Pei Y., Li X., Zhu Y., Ge X., Sun Y., Liu N. (2019). Gh ABP19, a novel germin-like protein from Gossypium hirsutum, plays an important role in the regulation of resistance to Verticillium and Fusarium wilt pathogens. Front. Plant Sci..

[15] Ding T., Su B., Chen X., Xie S., Gu S., Wang Q., Huang D., Jiang H. (2017). An Endophytic Bacterial Strain Isolated from Eucommia ulmoides Inhibits Southern Corn Leaf Blight. Front. Microbiol..

[16] Zhou Q., Gu S.Y., Xu L., Tan G.J. (2019). Effects of endophyte DZSY21 colonization on DNA methylation levels in Maize. J. Hefei Norm. Univ..

[17] Bari R., Jones J. (2009). Role of plant hormones in plant defence responses. Plant Mol. Biol..

[18] Dickman M.B., Fluhr R. (2013). Centrality of Host Cell Death in Plant-Microbe Interactions. Annu. Rev. Phytopathol..

[19] Clarke J.D., Volko S.M., Ledford H., Dong A.X. (2000). Roles of salicylic acid, jasmonic acid, and ethylene in cpr-induced resistance in *Arabidopsis*. Plant Cell.

[20] Paradis E., Claude J., Strimmer K. (2004). APE: Analyses of Phylogenetics and Evolution in Rlanguage. Bioinformatics.

[21] Hang T., Ling X., He C., Xie S., Ding T. (2021). Isolation of the zmers4 gene from maize and its functional analysis in transgenic plants. Front. Microbiol..

[22] Zeng H.H. (2016). Chitosan Oligosaccharide-Induced Resistance to Pseudomonas syringaepv Tomato DC3000 in Arabidopsis and a Preliminary Study on the Resistance Mechanism.

[23] Gao X.N. (2012). Control of Sclerotinia sclerotiorum in Rapeseed by Endophytic Bacterial Strain Em7.

[24] Katagiri F., Thilmony R., He S.Y. (2002). The *Arabidopsis thaliana*-Pseudomonas syringae interaction. Arab. Book.

[25] Zha K., Xie H., Ge M., Wang Z., Wang Y., Si W., Gu L. (2019). Expression of Maize MADS Transcription Factor ZmES22 Negatively Modulates Starch Accumulation in Rice Endosperm. Int. J. Mol. Sci..

[26] Livak K.J., Schmittgen T.D. (2001). Analysis of relative gene expression data using realtime quantitative PCR and the 2-DDCt method. Methods.

[27] Nelson B., Cai X., Nebenfuhr A. (2007). A multicolored set of in vivo organelle markers for co-localization studies in *Arabidopsis* and other plants. Plant J..

[28] Bhadauria V., Miraz P., Kennedy R., Banniza S., Wei Y. (2010). Dual trypan-aniline blue fluorescence staining methods for studying fungus-plant interactions. Biotech. Histochem..

[29] Banerjee J., Maiti M.K. (2010). Functional role of rice germin-like protein 1 in regulation of plant height and disease resistance. Biochem. Biophys. Res. Commun..

[30] Banerjee J., Das N., Dey P., Maiti M.K. (2010). Transgenically expressed rice germin-like protein1 in tobacco causes hyper-accumulation of H_2_O_2_ and reinforcement of the cell wall components. Biochem. Biophys. Res. Commun..

[31] Ilyas M., Rasheed A., Mahmood T. (2016). Functional characterization of germin and germin-like protein genes in various plant species using transgenic approaches. Biotechnol. Lett..

[32] Feys B.J.F., Benedetti C.E., Turner P.J.G. (1994). *Arabidopsis* mutants selected for resistance to the phytotoxin coronatine are male sterile, insensitive to methyl jasmonate, and resistant to a bacterial pathogen. Plant Cell.

[33] Kloek A.P., Verbsky M.L., Sharma S.B., Schoelz J.E., Vogel J., Klessig D.F., Kunkel B.N. (2001). Resistance to Pseudomonas syringae conferred by an *Arabidopsis* thaliana coronatine-insensitive (coi1) mutation occurs through two distinct mechanisms. Plant J..

[34] de Torres Zabala M., Zhai B., Jayaraman S., Eleftheriadou G., Winsbury R., Yang R., Truman W., Tang S., Smirnoff N., Grant M. (2016). Novel JAZ co-operativity and unexpected JA dynamics underpin *Arabidopsis* defence responses to Pseudomonas syringae infection. New Phytol..

[35] Zhao X.Y., Qi C.H., Jiang H., Zheng P.F., Hao Y.J. (2018). Functional identification of apple on *MdHIR4* in biotic stress. Plant Sci..

[36] Liu M., Khan N.U., Wang N., Yang X., Qiu D. (2016). The protein elicitor PevD1 enhances resistance to pathogens and promotes growth in *Arabidopsis*. Int. J. Biol. Sci..

[37] Averyanov A. (2008). Oxidative burst and plant disease resistance. Front. Biosci..

[38] Munir F., Hayashi S., Batley J., Naqvi S.M.S., Mahmood T. (2016). Germin-like protein 2 gene promoter from rice is responsive to fungal pathogens in transgenic potato plants. Funct. Integr. Genom..

[39] Li Q.Y. (2019). The 919-Position Glutamate of RBOHD Regulates the Molecular Mechanism of Lipopolysaccharide-Induced Reactive Oxygen Species Production.

[40] Li P., Zhao L., Qi F., Htwe N.M., Li Q., Zhang D., Lin F., Shang-Guan K., Liang Y. (2021). The receptor-like cytoplasmic kinase ripk regulates broad-spectrum ros signaling in multiple layers of plant immune system. Mol. Plant.

[41] Dickman M.B., Park Y.K., Oltersdorf T., Li W., Clemente T., French R. (2001). Abrogation of disease development in plants expressing animal antiapoptotic genes. Proc. Natl. Acad. Sci. USA.

[42] Eri M.G., Alex L. (2000). The hypersensitive response facilitates plant infection by the necrotrophic pathogen botrytis cinerea. Curr. Biol..

[43] Elad Y., Williamson B., Tudzynski P., Delen N. (2004). Botrytis: Biology, Pathology, and Control.

