# Supplementary material for: ZmGLP1, a Germin-like Protein from Maize, Plays an Important Role in the Regulation of Pathogen Resistance
Source: Int J Mol Sci. 2022 Nov 18;23(22):14316. doi: 10.3390/ijms232214316 (PMC9699084; doi:10.3390/ijms232214316)
Supplement: Supplementary file 1 [file ijms-23-14316-s001.zip › ijms-1930704-SI.pdf]

Supplementary Figures and Tables Abstract

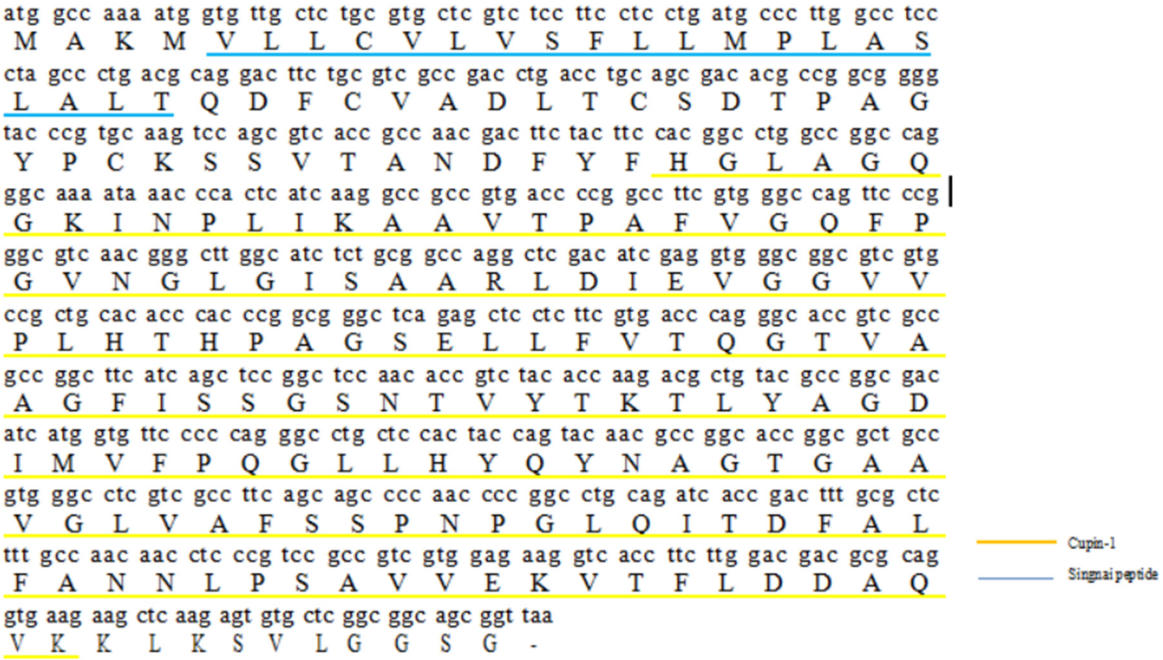

Figure S1. Molecular characterization of *ZmGLP1*.

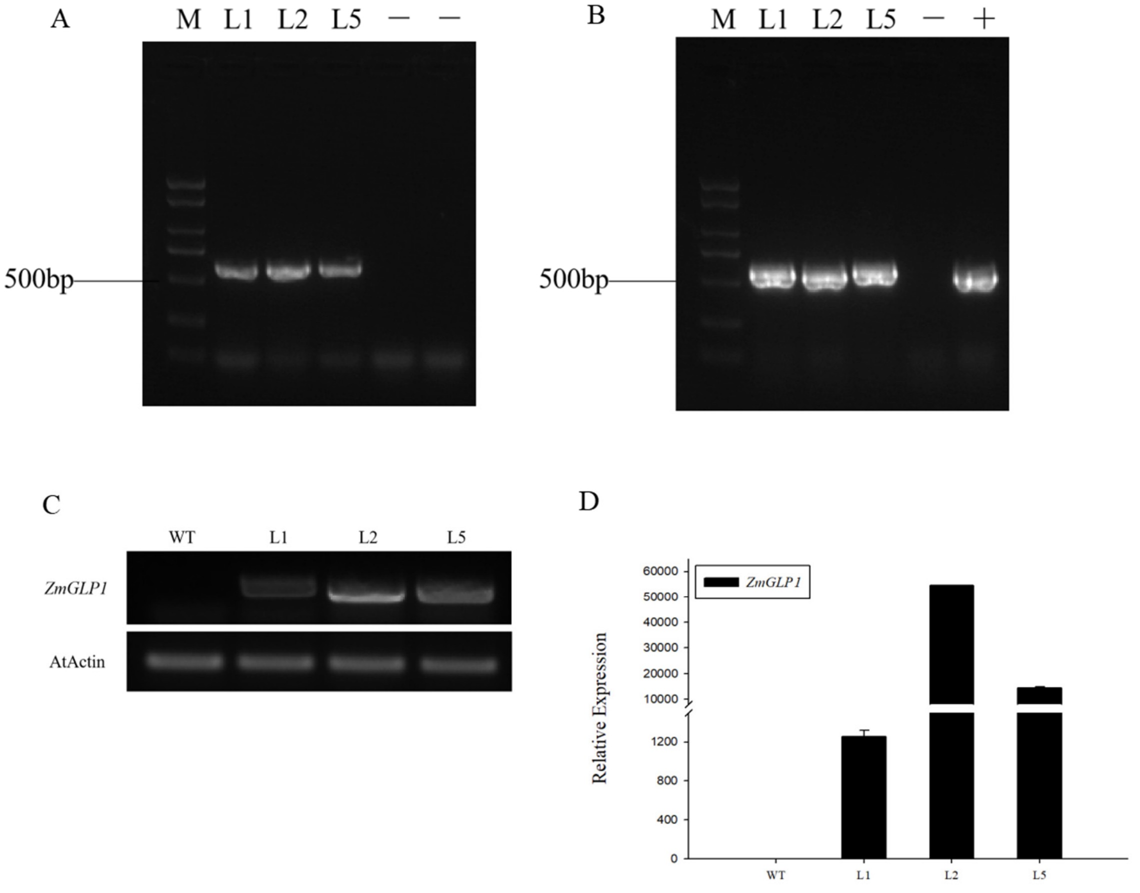

Figure S2. Detection of *ZmGLP1* transgenic *Arabidopsis thaliana*. (A) PCR analysis of *ZmGLP1*. (B) PCR analysis of GUS. M: 2K DNA marker; +: Positive control; -: Negative control. (C) The expression

of *ZmGLP1* gene in T3 transgenic *Arabidopsis thaliana* was detected by semi-quantitative PCR. (D) qRT-PCR analysis of *ZmGLP1* gene expression in T3 transgenic *Arabidopsis thaliana* line.

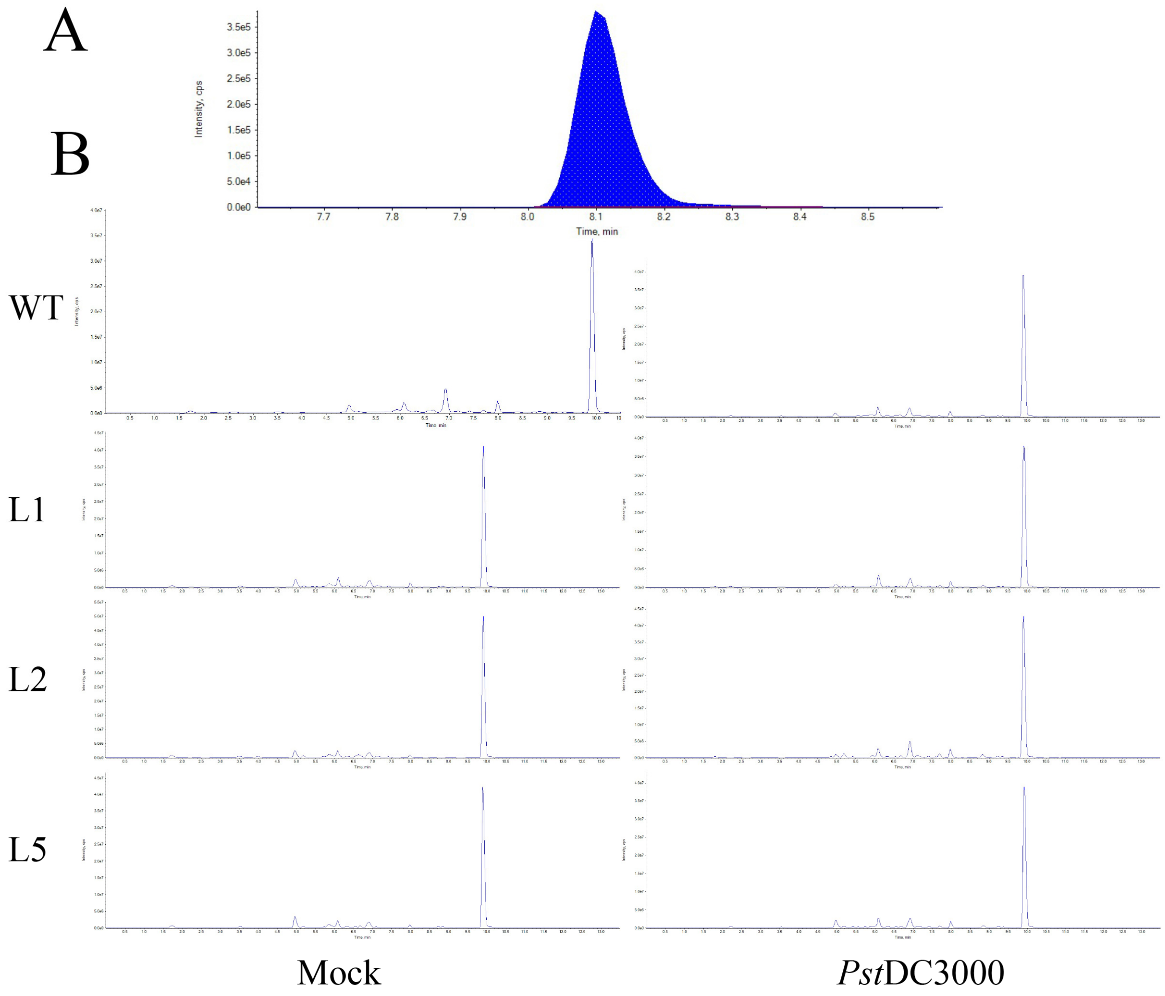

Figure S3. Chromatogram of JA extracted from *Arabidopsis*. (A) JA chromatogram of standard JA. (B) JA chromatogram of Wild-type (Col-0) and transgenic *Arabidopsis* L1 and L2 and L5 infected with *Pst*DC3000.

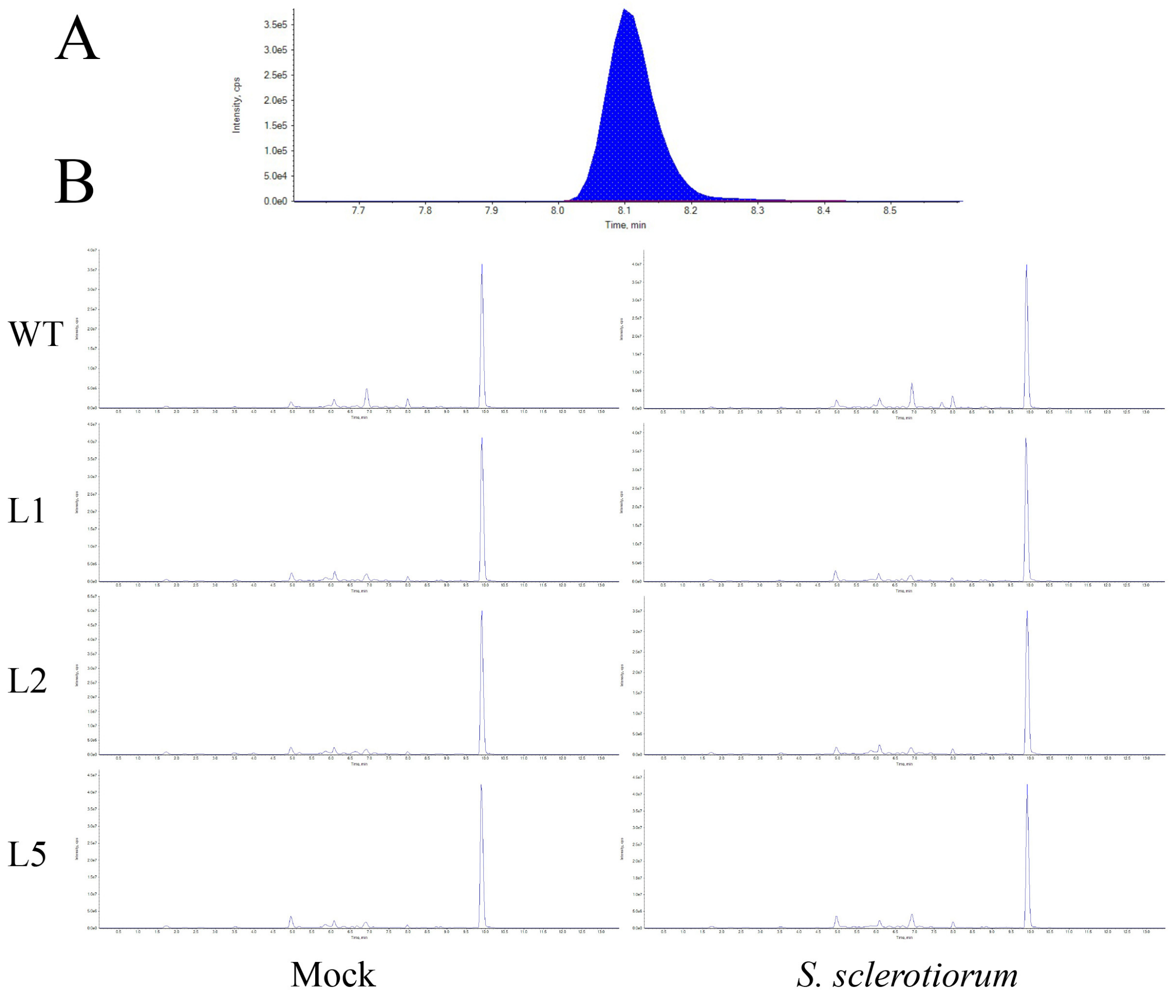

Figure S4. Chromatogram of JA extracted from *Arabidopsis*. (A) JA chromatogram of standard JA. (B) JA chromatogram of Wild-type (Col-0) and transgenic *Arabidopsis* L1 and L2 and L5 infected with *Sclerotinia sclerotiorum*.

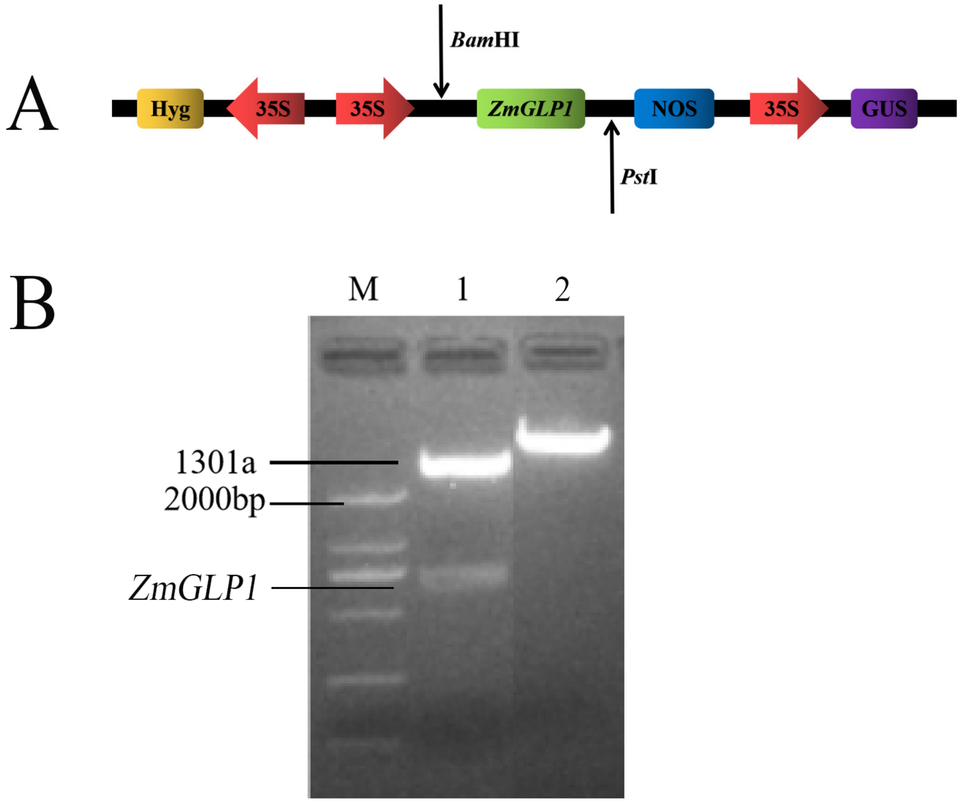

Figure S5. Construction of the p1301-*ZmGLP1* vector. (A) The pCAMBIA1301 vector included a CaMV35S promoter, Hyg and GUS tags. (B) Electrophoretic map of double enzyme digestion validation. M: 2K DNA marker; 1: p1301a-*ZmGLP1* recombinant plasmid with restriction digestion. The top band was 1301a plasmid with 12837bp, and the bottom band was *ZmGLP1* with 639bp; 2: p1301a-*ZmGLP1* recombinant plasmid without restriction digestion.

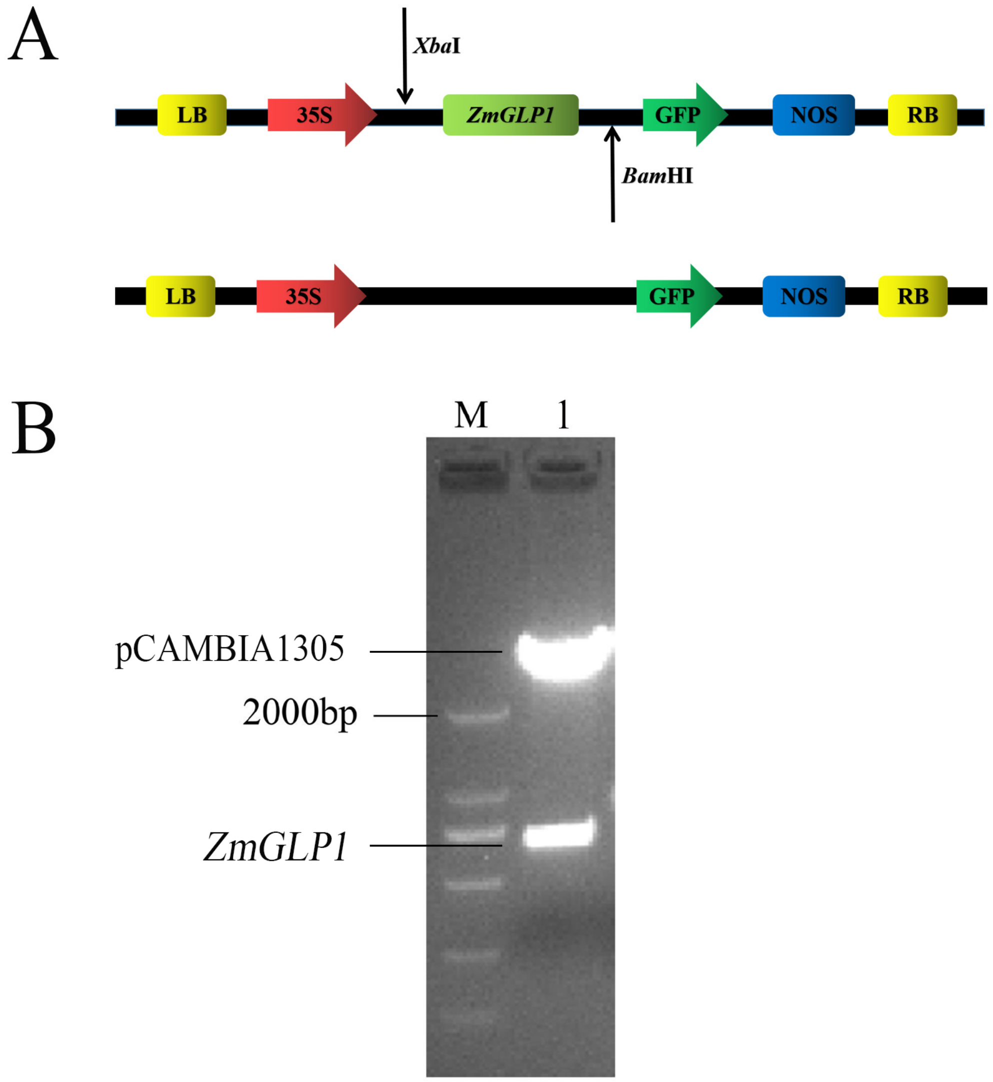

Figure S6. Construction of the p1305-*ZmGLP1*-GFP vector. (A) The pCAMBIA1305 vector included a CaMV35S promoter and GFP tag; *ZmGLP1* was inserted in front of the GFP. (B) Electrophoretic map of double enzyme digestion validation. M: 2K DNA marker; 1: The top band was p1305plasmid with 12680bp, and the bottom band was *ZmGLP1* with 639bp.

Table S1. *GLP* gene family in *Oryza sativa* and *Arabidopsis* and *Maize*.

Gene Name	Locus	Species
OsGLP1-1	LOC_Os01g14670.1	*Oryza sativa*
OsGLP1-2	LOC_Os01g18170.1	*Oryza sativa*
OsGLP1-3	LOC_Os01g50900.1	*Oryza sativa*
OsGLP1-4	LOC_Os01g72290.1	*Oryza sativa*
OsGLP1-5	LOC_Os01g72300.1	*Oryza sativa*
OsGLP2-1	LOC_Os02g29000.1	*Oryza sativa*
OsGLP2-2	LOC_Os02g29010.1	*Oryza sativa*
OsGLP2-3	LOC_Os02g29020.1	*Oryza sativa*
OsGLP2-4	LOC_Os02g32980.1	*Oryza sativa*

OsGLP3-1	LOC_Os03g08150.1	*Oryza sativa*
OsGLP3-2	LOC_Os03g44880.1	*Oryza sativa*
OsGLP3-3	LOC_Os03g48750.1	*Oryza sativa*
OsGLP3-4	LOC_Os03g48760.1	*Oryza sativa*
OsGLP3-5	LOC_Os03g48770.1	*Oryza sativa*
OsGLP3-6	LOC_Os03g48780.1	*Oryza sativa*
OsGLP3-7	LOC_Os03g58980.1	*Oryza sativa*
OsGLP3-8	LOC_Os03g59010.1	*Oryza sativa*
OsGLP3-9	LOC_Os03g58990.1	*Oryza sativa*
OsGLP4-1	LOC_Os04g52720.1	*Oryza sativa*
OsGLP5-1	LOC_Os05g10830.1	*Oryza sativa*
OsGLP5-2	LOC_Os05g19670.1	*Oryza sativa*
OsGLP8-1	LOC_Os08g08920.1	*Oryza sativa*
OsGLP8-2	LOC_Os08g08960.1	*Oryza sativa*
OsGLP8-3	LOC_Os08g08970.1	*Oryza sativa*
OsGLP8-4	LOC_Os08g08980.1	*Oryza sativa*
OsGLP8-5	LOC_Os08g08990.1	*Oryza sativa*
OsGLP8-6	LOC_Os08g09000.1	*Oryza sativa*
OsGLP8-7	LOC_Os08g09010.1	*Oryza sativa*
OsGLP8-8	LOC_Os08g09020.1	*Oryza sativa*
OsGLP8-9	LOC_Os08g09040.1	*Oryza sativa*
OsGLP8-10	LOC_Os08g09060.1	*Oryza sativa*
OsGLP8-11	LOC_Os08g09080.1	*Oryza sativa*
OsGLP8-12	LOC_Os08g13440.1	*Oryza sativa*
OsGLP8-13	LOC_Os08g35750.1	*Oryza sativa*
OsGLP8-14	LOC_Os08g35760.1	*Oryza sativa*
OsGLP9-1	LOC_Os09g39510.1	*Oryza sativa*
OsGLP9-2	LOC_Os09g39520.1	*Oryza sativa*
OsGLP9-3	LOC_Os09g39530.1	*Oryza sativa*
OsGLP11-1	LOC_Os11g33110.1	*Oryza sativa*
OsGLP12-1	LOC_Os12g05840.1	*Oryza sativa*
OsGLP12-2	LOC_Os12g05860.1	*Oryza sativa*
OsGLP12-3	LOC_Os12g05870.1	*Oryza sativa*
OsGLP12-4	LOC_Os12g05880.1	*Oryza sativa*
AtGLP1-1	AT1G02335.1	*Arabidopsis thaliana*
AtGLP1-2	AT1G09560.1	*Arabidopsis thaliana*
AtGLP1-3	AT1G10460.1	*Arabidopsis thaliana*
AtGLP1-4	AT1G18970.1	*Arabidopsis thaliana*
AtGLP1-5	AT1G18980.1	*Arabidopsis thaliana*
AtGLP1-6	AT1G72610.1	*Arabidopsis thaliana*
AtGLP1-7	AT1G74820.1	*Arabidopsis thaliana*
AtGLP3-1	AT3G04150.1	*Arabidopsis thaliana*
AtGLP3-2	AT3G04170.1	*Arabidopsis thaliana*

AtGLP3-3	AT3G04180.1	*Arabidopsis thaliana*
AtGLP3-4	AT3G04190.1	*Arabidopsis thaliana*
AtGLP3-5	AT3G04200.1	*Arabidopsis thaliana*
AtGLP3-6	AT3G05930.1	*Arabidopsis thaliana*
AtGLP3-7	AT3G05950.1	*Arabidopsis thaliana*
AtGLP3-8	AT3G10080.1	*Arabidopsis thaliana*
AtGLP3-9	AT3G62020.1	*Arabidopsis thaliana*
AtGLP4-1	AT4G14630.1	*Arabidopsis thaliana*
AtGLP5-1	AT5G20630.1	*Arabidopsis thaliana*
AtGLP5-2	AT5G26700.1	*Arabidopsis thaliana*
AtGLP5-3	AT5G38910.1	*Arabidopsis thaliana*
AtGLP5-4	AT5G38930.1	*Arabidopsis thaliana*
AtGLP5-5	AT5G38940.1	*Arabidopsis thaliana*
AtGLP5-6	AT5G38960.1	*Arabidopsis thaliana*
AtGLP5-7	AT5G39100.1	*Arabidopsis thaliana*
AtGLP5-8	AT5G39110.1	*Arabidopsis thaliana*
AtGLP5-9	AT5G39120.1	*Arabidopsis thaliana*
AtGLP5-10	AT5G39130.1	*Arabidopsis thaliana*
AtGLP5-11	AT5G39150.1	*Arabidopsis thaliana*
AtGLP5-12	AT5G39160.1	*Arabidopsis thaliana*
AtGLP5-13	AT5G39180.1	*Arabidopsis thaliana*
AtGLP5-14	AT5G39190.1	*Arabidopsis thaliana*
AtGLP5-15	AT5G61750.1	*Arabidopsis thaliana*
ZmGLP1-1	Zm00001d033583	*Zea mays*
ZmGLP1-2	Zm00001d032047	*Zea mays*
ZmGLP1-3	Zm00001d034413	*Zea mays*
ZmGLP1-4	Zm00001d033447	*Zea mays*
ZmGLP1-5	Zm00001d032048	*Zea mays*
ZmGLP1-6	Zm00001d029062	*Zea mays*
ZmGLP1-7	Zm00001d034514	*Zea mays*
ZmGLP1-8	Zm00001d027890	*Zea mays*
ZmGLP1-9	Zm00001d005333	*Zea mays*
ZmGLP1-10	Zm00001d035700	*Zea mays*
ZmGLP6-1	Zm00001d038137	*Zea mays*
ZmGLP1	Zm00001d037513	*Zea mays*
ZmGLP6-3	Zm00001d035597	*Zea mays*
ZmGLP8-1	Zm00001d011036	*Zea mays*
ZmGLP7-1	Zm00001d018954	*Zea mays*
ZmGLP8-2	Zm00001d008210	*Zea mays*
ZmGLP9-1	Zm00001d048176	*Zea mays*
ZmGLP8-3	Zm00001d011853	*Zea mays*
ZmGLP9-2	Zm00001d048219	*Zea mays*
ZmGLP10-1	Zm00001d025059	*Zea mays*

ZmGLP10-2	Zm00001d000006	*Zea mays*
ZmGLP10-3	Zm00001d000008	*Zea mays*
ZmGLP10-4	Zm00001d000012	*Zea mays*
ZmGLP10-5	Zm00001d000017	*Zea mays*
ZmGLP10-6	Zm00001d000018	*Zea mays*
ZmGLP5-1	Zm00001d016697	*Zea mays*
ZmGLP5-2	Zm00001d015347	*Zea mays*
ZmGLP4-1	Zm00001d053249	*Zea mays*
ZmGLP4-2	Zm00001d049438	*Zea mays*
ZmGLP4-3	Zm00001d049428	*Zea mays*
ZmGLP4-4	Zm00001d049432	*Zea mays*
ZmGLP4-5	Zm00001d052654	*Zea mays*
ZmGLP4-6	Zm00001d049436	*Zea mays*
ZmGLP4-7	Zm00001d049431	*Zea mays*
ZmGLP4-8	Zm00001d049434	*Zea mays*
ZmGLP4-9	Zm00001d049425	*Zea mays*
ZmGLP4-10	Zm00001d050446	*Zea mays*
ZmGLP4-11	Zm00001d049430	*Zea mays*
ZmGLP4-12	Zm00001d049439	*Zea mays*
ZmGLP4-13	Zm00001d049424	*Zea mays*
ZmGLP4-14	Zm00001d049433	*Zea mays*
ZmGLP4-15	Zm00001d049423	*Zea mays*
ZmGLP4-16	Zm00001d049420	*Zea mays*
ZmGLP4-17	Zm00001d049418	*Zea mays*
ZmGLP4-18	Zm00001d049427	*Zea mays*
ZmGLP4-19	Zm00001d049435	*Zea mays*
ZmGLP4-20	Zm00001d049426	*Zea mays*
ZmGLP3-1	Zm00001d043710	*Zea mays*
ZmGLP4-21	Zm00001d049419	*Zea mays*
ZmGLP4-22	Zm00001d049421	*Zea mays*
ZmGLP4-23	Zm00001d049437	*Zea mays*
ZmGLP3-2	Zm00001d040519	*Zea mays*
ZmGLP3-3	Zm00001d040997	*Zea mays*
ZmGLP2-1	Zm00001d002295	*Zea mays*
ZmGLP2-2	Zm00001d004401	*Zea mays*
ZmGLP2-3	Zm00001d003801	*Zea mays*

Table S2. Primers of qRT-PCR used in this study.

Primers name	Forward primer(5′→3′)	Reverse primer(5′→3′)
qZmActin1	GGGATTGCCGATCGTATGAG	GAGCCACCGATCCAGACACT
qZmGADPH	CTTCGGCATTGTTGAGGGTTTG	TCCTTGGCTGAGGGTCCGTC
qZmGLP1	TCTCCTTCCTCCTGATGCCC	GCGGTGACGCTGGACTTGC

qAtActin2	GGTAACATTGTGCTCAGTGGTGG	AACGACCTTAATCTTCATGCT TGC
qAtTUB4	CGAAAACGCTGACGAGTGTA	CCTTGGGAATGGGATAAGGT
qAtPR1	CCTTACGGGGAAAACTTAGCCT	CCGAGTCTCACTGACTTTCTCC
qAtPR5	AACGGTAGATGTGTAACCGGAG	CGATCCTCCGGATGGTCTTATC
qICS1	GCTTGGCTAGCACAGTTACAGC	CACTGCAGACACCTAATTGAGTCC
qLOX2	ATGAGCCTGTTATCAATGCTGC	AACACCAGCTCCAGCTCTATTCTT
qLOX3	ATGAGCCTGTTATCAATGCTGC	AACACCAGCTCCAGCTCTATTCTT
qAOS	TCGCCGAGAATCCACAAGTC	GTAGCCTCCGGTTAGTTCCG
qAOC1	AGGTTCCTCCTCTCCGACAA	AACTTTGCTTGGTCTGGGGT
qVSP2	TGACCGTTGGAAGTTGTGGA	CGAACCATTAGGCTTCAATATGAGA
qERF1	ACCGCTCCGTGAAGTTAGATAATG	ATCCTAATCTTTCACCAAGTCCCAC
qERF2	GCTTTTAGGATGCGTGGTTC	CTCCTTCGTTTCAACTTCCC
